# COMMD10 inhibited DNA damage to promote the progression of gastric cancer

**DOI:** 10.1007/s00432-024-05817-z

**Published:** 2024-06-13

**Authors:** Xiaohua Liu, Xiaocheng Mao, Chao Zhu, Hongfei liu, Yangyang Fang, Tianmei Fu, Linwei Fan, Mengwei Liu, Ziqing Xiong, Hong Tang, Piaoping Hu, Aiping Le

**Affiliations:** Department of Blood Transfusion, Key Laboratory of Jiangxi Province for Transfusion Medicine, The First Affiliated Hospital, Jiangxi Medical College, Nanchang University, 1519 Dongyue Avenue, Nanchang, Jiangxi People’s Republic of China

**Keywords:** COMMD10, Gastric cancer, ATM-p53, DNA damage repair

## Abstract

**Purpose:**

The copper metabolism MURR1 domain 10 (COMMD10) plays a role in a variety of tumors. Here, we investigated its role in gastric cancer (GC).

**Methods:**

Online prediction tools, quantitative real-time PCR, western blotting and immunohistochemistry were used to evaluate the expression of COMMD10 in GC. The effect of COMMD10 knockdown was investigated in the GC cell lines and in in vivo xenograft tumor experiments. Western blotting and immunofluorescence were used to explore the relationships between COMMD10 and DNA damage.

**Results:**

The expression of COMMD10 was upregulated in GC compared to that in para-cancerous tissue and correlated with a higher clinical TNM stage (*P* = 0.044) and tumor size (*P* = 0.0366). High COMMD10 expression predicted poor prognosis in GC. Knockdown of COMMD10 resulted in the suppression of cell proliferation, migration, and invasion, accompanied by cell cycle arrest and an elevation in apoptosis rate. Moreover, the protein expression of COMMD10 was decreased in cisplatin-induced DNA-damaged GC cells. Suppression of COMMD10 impeded DNA damage repair, intensified DNA damage, and activated ATM–p53 signaling pathway in GC. Conversely, restoration of COMMD10 levels suppressed DNA damage and activation of the ATM-p53 signaling cascade. Additionally, knockdown of COMMD10 significantly restrained the growth of GC xenograft tumors while inhibiting DNA repair, augmenting DNA damage, and activating the ATM–p53 signaling pathway in xenograft tumor tissue.

**Conclusion:**

COMMD10 is involved in DNA damage repair and maintains genomic stability in GC; knockdown of COMMD10 impedes the development of GC by exacerbating DNA damage, suggesting that COMMD10 may be new target for GC therapy.

**Supplementary Information:**

The online version contains supplementary material available at 10.1007/s00432-024-05817-z.

## Introduction

Gastric cancer (GC) had been responsible for 1 million new cases and 768,793,000 deaths in 2020, according to the 2022 Global Cancer Statistics (Siegel et al. 2022). Despite advances in the treatment of GC, recurrence and progression remain common (Joshi and Badgwell 2021). As the exact mechanisms of gastric carcinogenesis and development remain largely unknown, urgent efforts are needed to unravel the mechanisms of GC development and identify novel therapeutic targets to improve the survival of patients with GC.

The copper metabolism MURR1 domain (COMMD) family was discovered in recent decades. It consists of 10 family members (COMMD1–COMMD10) and is characterized by the presence of a highly conserved and unique motif named the COMM domain at the extreme carboxyl termini of the proteins (Maine and Burstein 2007), which serves as an interface for COMMD–COMMD protein interactions (Burstein et al. 2005). These members are highly conserved throughout evolution and share certain functional properties including the regulation of transcription factor NF-κB activity (Maine and Burstein 2007), immune response (Shirai et al. 2023), copper homeostasis (Corbee and Penning 2021), endosomal sorting (Healy et al. 2023), and epithelial sodium channel function (Maine and Burstein 2007). In recent years, the COMMD family of proteins has been shown to function in various tumors (Suraweera et al. 2021a, b, 2020; Zhan et al. 2017; Zheng et al. 2019; Tang et al. 2023; Yang et al. 2017, 2021, 2022). However, the role of the COMMD family members in GC has not been explored. COMMD10 was shown to inactivate NF-κB signaling pathway in colorectal cancer (Yang et al. 2017) and hepatocellular carcinoma (Yang et al. 2021), thereby inhibiting the development of these tumors. Moreover, COMMD10 can enhance ferroptosis by disturbing the balance of Cu and Fe, thus increasing the radiosensitivity of hepatocellular carcinoma (Yang et al. 2022). In addition, COMMD10 is critical for liver Kupffer cell function (Cohen et al. 2021; Ben Shlomo et al. 2019). Kyoto Encyclopedia of Genes and Genomes (KEGG) enrichment analysis of COMMD10 and its and its interacted genes showed that they were significantly enriched in pathways in cancer, hypoxia-inducible factor signaling, ubiquitin-mediated proteolysis, endocytosis, renal cell carcinoma and mineral uptake (Fan et al. 2020). However, the study remains at the level of bioinformatics analysis and expression profiling, and further experimental validation is required. Overall, additional functions of COMMD10, as well as its role and mechanism in GC development, remain to be explored.

Cells encounter thousands of DNA damage events every day(Waterman et al. 2020). Among these, DNA double-strand breaks (DSBs) are the most lethal, and improper repair can lead to chromosomal breaks and cell death. In response to the threat posed by DNA damage, eukaryotes have evolved the DNA damage response (DDR), which is characterized by DNA repair and cell cycle arrest to maintain genomic stability (Groelly et al. 2023). Ataxia telangiecasia mutated (ATM) (Lee and Paull 2021) is the most upstream DDR kinase that recognizes DNA damage and transmits the damage signal to downstream target proteins such as p53, a well-known tumor suppressor gene that plays a crucial role in the regulation of cell cycle, apoptosis, and senescence (Shiloh 2003; Lavin 2008; Bakkenist and Kastan 2003; Roos et al. 2016). Phosphorylated H2A histone family member X (γH2AX) is widely used as a sensitive biomarker of DNA damage (Siddiqui et al. 2015). There are two pathways to repair DNA DSBs, homologous recombination and non-homologous end joining (Khanna and Jackson 2001), in which a number of repair proteins are involved, such as RAD51,BRCA1 (Khanna and Jackson 2001), XRCC4, DNA ligase 4 (LIG4), Ku70, and Ku80 (Zhao et al. 2020). If the damage is successfully repaired, DDR inactivation occurs and normal cellular function is restored. Once repair fails, DDR signaling triggers cell death via apoptosis or cellular senescence (Jackson and Bartek 2009). Although DNA repair pathways maintain genomic stability in normal cells and prevent cancer onset, once a tumor has arisen, the same DNA repair pathways can be harnessed therapeutically to kill cancer cells. This makes the inhibition of DNA repair proteins in cancer cells an advantageous anti-tumor therapy (Suraweera et al. 2021a). COMMD1 (Suraweera et al. 2021a) and COMMD4 (Suraweera et al. 2021b; Tang et al. 2023), two members of COMMD family, were reported to participate in DNA damage repair to maintain genomic stability in non-small cell lung cancer. H2B peptide directly binds to COMMD4 and inhibits its interactions, thereby increasing sensitivity to radiotherapy by causing DNA DSBs accumulation and mitotic catastrophe, suggesting COMMD proteins are potential targets for cancer therapy (Tang et al. 2023). This suggests that the COMMD family may be associated with DNA damage and a potential target for anti-tumor therapy.

In this study, we analyzed the expression and prognostic differences of the COMMD family in GC using The Cancer Genome Atlas (TCGA) database and found that COMMD10 was highly expressed and associated with poor prognosis in GC. Knockdown of COMMD10 suppressed cell proliferation, migration, and invasion, caused cell cycle arrest and increased the apoptosis rate in GC. Additionally, COMMD10 was implicated in the repair of DNA damage in GC cells. Suppression of COMMD10 resulted in impaired DNA repair, enhanced DNA damage, and activated of the ATM-p53 signaling pathway in GC. Overall, knockdown of COMMD10 hinders the progression of GC by exacerbating DNA damage, offering a potential novel therapeutic strategy for GC treatment.

## Materials and methods

### Bioinformatics analysis and database

RNA-sequencing expression profiles for 375 GC samples and 391 normal samples were downloaded from a TCGA dataset (https://portal.gdc.com) to analyze the expression and prognosis of COMMD family members in GC. Kaplan–Meier survival analysis was conducted to compare the survival probability between the high and low COMMD expression groups. COMMD10 mRNA expression data were downloaded from TCGA (https://cancergenome.nih.gov/) and the Genotype-Tissue Expression (GTEx) databases to verify the expression of COMMD10 in GC. Gene Expression Profiling Interactive Analysis (GEPIA) (https://GEPIA2(cancer-pku.cn) was used to confirm the prognostic differences between high COMMD10 (n = 192) and low COMMD10 (n = 191) expression groups in GC, and to investigate the expression correlations between COMMD10 and DNA repair proteins.

### Patients and tissue specimens

Seventy-one pairs of paraffin-embedded GC specimens from untreated patients who underwent surgical resection at the First Affiliated Hospital of Nanchang University between 2018 and 2021 were collected. The clinicopathological characteristics of the patients are shown in Table 1. This study obtained ethics committee approvement from the hospital and complied with the Declaration of Helsinki. Written informed consent was obtained from all the patients.

## Cell culture

The immortalized human gastric epithelial cell line, GES-1, and three gastric cancer cell lines, MKN45, HGC27, and MGC803, were obtained from the Chinese Medical Science Academy’s Cell Culture Center (Shanghai, China). All cells were cultured in RPMI-1640 (Gibco, Gaithersburg, MD) supplemented with 10% fetal bovine serum (FBS; Gibco) and maintained in an incubator at 37 °C under an atmosphere of 5% CO_2_.

### RNA extraction and quantitative real-time PCR (qPCR)

Total RNA from the cells was extracted using TRIzol reagent (Absin, Shanghai, China), and then the extracted RNA was reverse transcribed into complementary DNA according to the manufacturer's instructions of a reverse transcription kit (TransGen Biotech, Beijing, China). Subsequently, qPCR was conducted by M5 HiPer Real-Time PCR Super mix (Mei5bio, Beijing, China). Primer sequences used in this study are listed below. COMMD10: forward-5′-GCT GAA GCA TTT GTC AAT ACG TGG-3′, reverse-5′-GCC ATC TGA AGG TTA AGC TGCC-3′; β-actin: forward-5-′CAC CAT TGG CAA TGA GCG GTTC-3′, reverse- 5′-AGG TCT TTG CGG ATG TCC ACGT-3′. β-actin was used as an internal control. 2^–ΔΔCT^ method was used to calculated the relative expression of RNA.

### Western blotting

Cell and tissue samples were collected and lysed at 4 °C using RIPA buffer (Solarbio, Beijing, China). The prepared protein samples were separated using SDS-PAGE and transferred onto nitrocellulose membranes (Millipore, MA, USA). After blocking with 5% skim milk powder at room temperature for 1 h, The membranes were incubated at 4 °C overnight with the primary antibody. Primary antibodies used in this study included anti-COMMD10 (1:1000, Abcam, Cambridge, USA), anti-GAPDH (1:10,000, Proteintech, Wuhan, Hubei, China), anti-phospho-p53 (Ser15) (1:1000, Proteintech), anti-Histone phospho-H2A.X (Ser139) (γ-H2AX, 1:1000, Cell Signaling Technology, Danvers, USA), anti-ATM (1:500, Zenbio, Chengdu, Sichuan, China), anti-phospho-ATM (Ser1981) (1:500, Zenbio), anti-RAD51 (1:1000, Proteintech), and anti-BRCA1 (1:500, Zenbio). The next day, the membranes were washed thrice with Tris-buffered saline containing Tween (TBST, Solarbio), and then incubated with HRP goat anti-rabbit IgG (1:5000, Proteintech) or HRP goat anti-mouse IgG (1:15,000, Proteintech) for 2 h at room temperature. Finally, the membrane was exposed to ECL reagent (NCM Biotech, Suzhou, China) to detect the target protein using a chemiluminescence imaging system (Analytik Jena, Jena, Germany).

### Immunohistochemistry (IHC)

After deparaffinization, rehydration, and blocking of tissue sections, primary antibodies (anti-COMMD10 antibody diluted 1:200, Abcam) were added and incubated overnight at 4 °C. The next day, after washing with phosphate-buffered saline (PBS, Solarbio), the sections were incubated with goat anti-rabbit IgG polyclonal antibody labeled with horseradish peroxidase (HRP, Zsbio, Beijing, China) for 30 min at room temperature. The tissue sections were stained with DAB and hematoxylin (Zsbio). The immunohistochemistry (IHC) scores of the specimens were determined based on the degree and intensity of staining. The median IHC score of the tissue was used to determine the target protein expression. Low expression was considered below the median, and vice versa.

### Transfection of plasmid

The sequences of scramble and shRNA targeting the human COMMD10 gene (shCOMMD10-1 and shCOMMD10-2) were as follows: (scramble: 5′-CCT AAG GTT AAG TCG CCC TCG -3′; shCOMMD10-1: 5′ -GCT TAT CAC TTC TTA GAC AAA -3′; shCOMMD10-2: 5′-GCA TTC TCT CTA GAG AAA CAA -3′. The MKN45, HGC27, and MGC803 GC cell lines were pre-seeded into six-well plates at a density of 1 × 10^5^ cells/well and then infected with lentivirus. After 12 h of infection, the virus-containing medium was replaced with normal medium and puromycin (2 mg/mL) was used to establish COMMD10 stable knockdown cell lines, which were used in subsequent cell function experiments.

Moreover, the COMMD10 overexpression plasmid was procured from GenePharma (Shanghai, China) and transiently transfected using jetPRIME transfection reagent (Polyplus, France). Prior to transfection, cells were pre-seeded in 6-well plates with a cell density maintained between 60 and 80%. On the subsequent day, both the vector and COMMD10 plasmid (2.5 μg each) were separately dissolved in 200 μL buffer, vortexed for 10 s, and then spun down. Subsequently, 5 μL of transfection reagent was added to each mixture followed by vortexing for 1 s and spinning down. The mixtures were left at room temperature for a duration of 10 min before being added to the cells. After an incubation period of 4–6 h, the medium was replaced with fresh medium and cultured for an additional 48 h to facilitate protein extraction or subsequent experiments. Furthermore, the COMMD10 overexpression plasmid was employed to restore the expression level of COMMD10 after knockdown in GC cells designated as COMMD10 ^RES^.

### Cell proliferation and colony formation assays

GC cells were seeded in cell culture plates at a density of 1 × 10^4^ per well and cultured for 7 days, with a medium change every 3 days. Subsequently, cell counts were performed on days 1, 3, 5, and 7, and the data were statistically analyzed.

For the colony formation assay, GC cells were seeded in 6-well plates at a density of 1,000 cells per well and cultured continuously for one week, with a medium change every 3 days. The cells were fixed with 4% methanol after the cell clones became visible, stained with crystal violet (Solarbio) and photographed. All the experiments were repeated three times.

### EdU staining assay

EdU staining assay was performed by EdU-594 Cell Proliferation Detection Kit (Beyotime). In brief, cells were pre-cultured in 6-well plates and incubated with 10 μM 5-ethynyl-2′-deoxyuridine (EdU) for 2 h. The cells were stained with Azide 594. The nuclei were then stained with 4’-6-diamidino-2-phenylindole (DAPI) for 10 min. Finally, EdU-positive cells were observed and photographed under a fluorescence microscope (Olympus, Tokyo, Japan).

### Transwell assay

The transwell chambers (Corning, New York, USA) used in invasion experiments were pre-coated with Matrigel gel (BD, New Jersey, USA). In migration assays, 600 μL medium containing 20% FBS was added to the lower chamber, and 200 μL transfected GC cells cultured with serum-free medium were seeded into the upper layer. Cells passing through the chambers were fixed with methanol, stained with crystal violet, and photographed. Each experiment was performed in triplicate.

### Cell cycle and apoptosis analysis

Cell cycle assays were performed using a Cell Cycle Analysis Kit (Beyotime). In brief, cells were collected, washed twice with PBS, and then fixed with pre-cooled 70% alcohol at 4 °C for 24 h. On the second day, the fixed cells were washed twice with cold PBS and then stained with PI, incubated at 37 °C in the dark for 30 min according to the manufacturer's instructions. Cell cycle distribution was determined using a FACS flow cytometer (BD Biosciences, San Jose, CA, US). The results were analyzed using Modifit software (version 5.0).

Apoptosis was detected using the Annexin V FITC Apoptosis Kit (BestBio, Shanghai, China). Cells were resuspended in 400 µL binding buffer, then 5 µL annexin V-FITC was added and incubated for 15 min at 4 °C in the dark, and then following the addition of 5 µL propidium iodide, were incubated for another 5 min in the dark; finally, the cells were analyzed by FACS flow cytometer. The analysis results were obtained using FlowJo 10.8.1 software.

### DNA damage induced by cisplatin

GC cells were pre-seeded in six-well plates, and 5 μM cisplatin (MCE, New Jersey, USA) was added to each group of cells the following day. After 24 h of incubation, cells were harvested and proteins were extracted for western blotting assays.

### Immunofluorescence

Cells were cultured in a confocal chamber, fixed in 4% paraformaldehyde (Beyotime) for 30 min, permeabilized with 0.1% Triton X-100 (Beyotime) for 15 min, and blocked with 5% BSA for 1 h at room temperature. After blocking, the cells were incubated with phosphor-H2AX-S139 (γ-H2AX, 1:200, Cell Signaling Technology) antibodies at 4 °C overnight. The following day, cells were incubated with Dylight488, donkey anti-rabbit IgG (H + L) (Earthox, San Francisco, USA) in the dark for 1 h at room temperature. DAPI was used to stain the nuclear DNA. Images were obtained by a confocal microscope (Carl Zeiss, Oberkochen, Germany).

### Comet assay

Cells were harvested and mixed with 0.5% low melting point agar, then spread evenly on slides pre-laid with 1% normal melting point agar gel, cooled for ten minutes at 4 °C, lysed in the dark at 4 °C for 1 h, then submerged in electrophoresis buffer for 30 min. Samples were electrophoresed for 30 min, stained with EB (YeaSen, Shanghai, China) solution, and visualized by confocal microscope (Carl Zeiss) to obtain an image. The percentage of DNA tails was estimated using the CASP software.

### Tumor xenografts experiments

Animal experiments were approved by the Institutional Animal Care and Use Committee of the First Affiliated Hospital of Nanchang University and were conducted in accordance with the guidelines and protocols for animal care and protection. Four-week-old female BALB/c nude mice (n = 10) were purchased from Hangzhou Ziyuan Laboratory Animal Technology (Hangzhou, China) and randomly divided into scramble (n = 5) and shCOMMD10 groups (n = 5). The concentration of MKN45 cells infected with shRNA lentivirus was adjusted to 4 × 10^6^ cells in 100 µL suspension and injected subcutaneously into the right forelimb of each mouse. Five days after injection, the tumor length and short diameter were measured and recorded every 3 days for 26 days. Subsequently, the tumor-bearing nude mice were sacrificed, the tumors were isolated, and their volume and weight measured. Finally, the tumor tissues were preserved for western blotting and IHC assays. Tumor volume was calculated using the formula *V* = (*L* × *W*^2^)/2, where *V*, *L*, and *W* represent the tumor volume, long diameter, and short diameter, respectively.

### Statistical analysis

Student's two-tailed *t*-test was used for the comparison between two groups while one-way analysis of variance (ANOVA) was used for the comparison between multiple groups. The statistical analysis was performed using Graphpad prism (10.0). The chi-square test was used to analyze statistical differences of clinicopathological characteristics in GC patients. Survival curves were plotted using the Kaplan–Meier method and analyzed by the log-rank test using SPSS 26.0 software. All data in this study were presented as mean ± SEM. A *p* < 0.05 was considered statistically significant.

## Results

### COMMD10 is upregulated and correlates with poor prognosis in GC

**Fig. 1:**
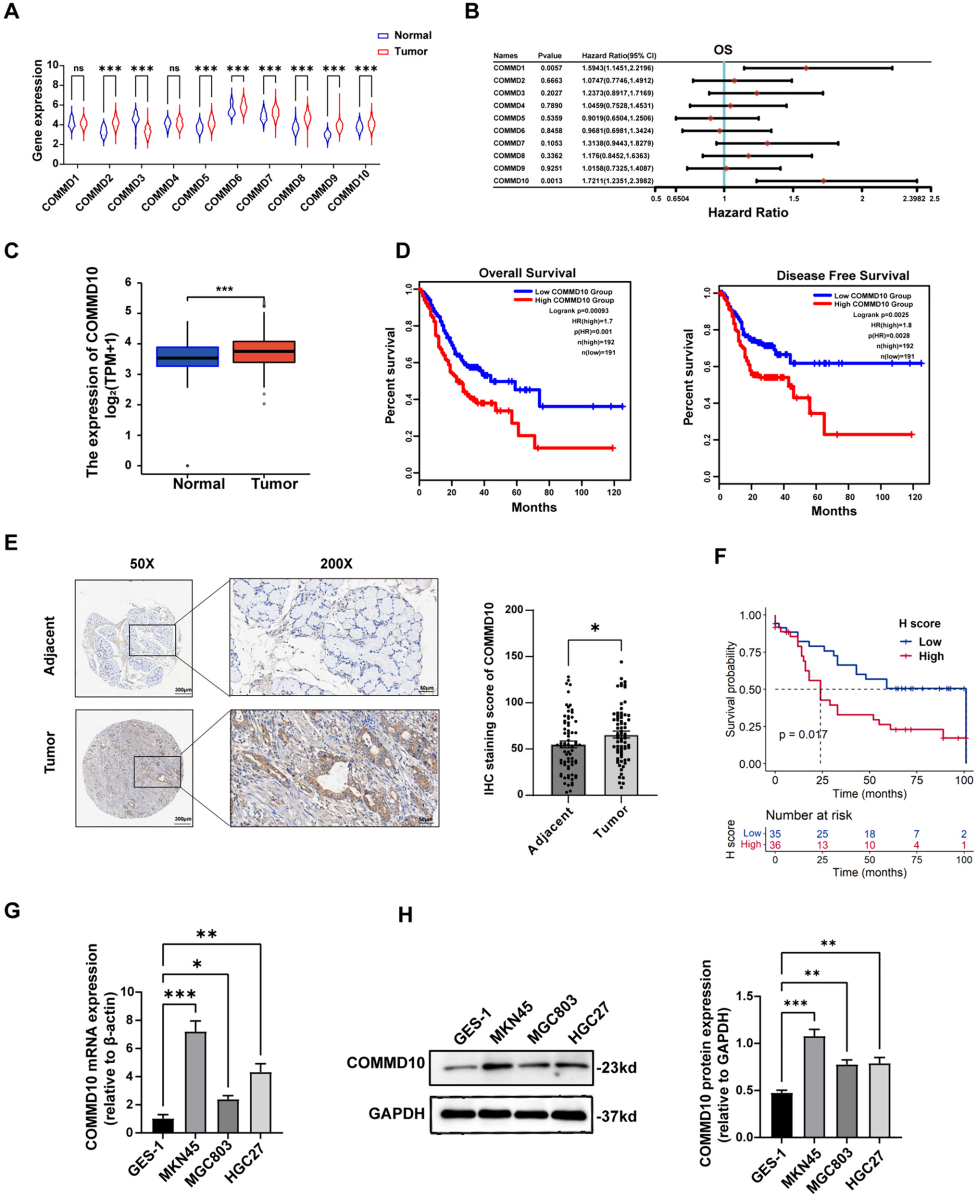
COMMD10 is upregulated in GC and predicted poor prognosis among GC patients. **A** Expressions of COMMD family in GC tissues (N = 375) and normal tissues (N = 391) based on Cancer Genome Atlas (TCGA) database (Wilcox test). **B** The overall survival of COMMD family in GC(N = 375) through TCGA database. **C** Differential expression of COMMD10 between GC (n = 375) and normal tissues (n = 32) from TCGA and Genotype-Tissue Expression (GTEx) database. **D** Overall survival and disease-free survival between high COMMD10 expression (N = 192) and low COMMD10 expression (N = 191) in GC obtained from Gene Expression Profiling Interactive Analysis (GEPIA). **E** Representative images of immunohistochemical staining of COMMD10 in 71 pairs of GC tissues and matched adjacent nontumor tissues. The IHC score of each sample is calculated and displayed in histogram. (two-tailed unpaired t test, *P < 0.05). **F** Kaplan‒Meier survival analysis of patients with higher COMMD10 expression levels (n = 35) and lower COMMD10 expression (n = 36) (log-rank test). **G** Quantitative real-time polymerase reaction (qPCR) and **H** Western blotting assays are performed to test the mRNA and protein levels of COMMD10 in GC cells. *P < 0.05, **P < 0.01, ***P < 0.001

Bioinformatics analysis from TCGA database was conducted to investigate the expression differences and prognosis of COMMD family in GC, the results showed that COMMD10 was upregulated in GC tissues (n = 375) compared to normal tissues (n = 391) (Fig. 1A). More importantly, prognostic analysis of the COMMD family in GC showed that COMMD10 was associated with poor prognosis, including overall survival (Fig. 1B), disease-free survival (Fig. S1A, B), disease-specific survival (Fig. S1C, D) and progression-free survival (Fig. S1E, F) in GC. Online tools were used to explore the expression difference and prognostic value of COMMD10 in GC. Similarly, COMMD10 was upregulated in GC (Fig. 1C), and patients with GC with higher COMMD10 expression had worse overall survival and progression-free survival (Fig. 1D) than in those with low expression. Therefore, we focused on the role of COMMD10 in GC development. First, a total of 71 pairs of GC tissues and their corresponding para-cancerous tissues were collected and stained by IHC to determine the expression of COMMD10. The clinical characteristics and overall survival of the patients were recorded and analyzed. As showed in Fig. 1E, the expression of COMMD10 was significantly higher in GC tissues than in adjacent tissues. In addition, COMMD10 expression correlated with higher clinical TNM stage (*P* = 0.044) and tumor size (*P* = 0.0366) (Table 1. Consistent with the prognostic results from the TCGA database, GC patients with higher COMMD10 expression had worse overall survival than those with low expression (Fig. 1F). Subsequently we also examined the expression of COMMD10 in GC cell lines and found that COMMD10 mRNA expression and protein levels were higher in GC cell lines than in the normal gastric epithelial cell line GES-1 (Fig. 1G, H). Taken together, our results suggest that COMMD10 expression is upregulated and is significantly associated with poor prognosis in GC.

### COMMD10 promotes the development of GC cells

**Fig. 2:**
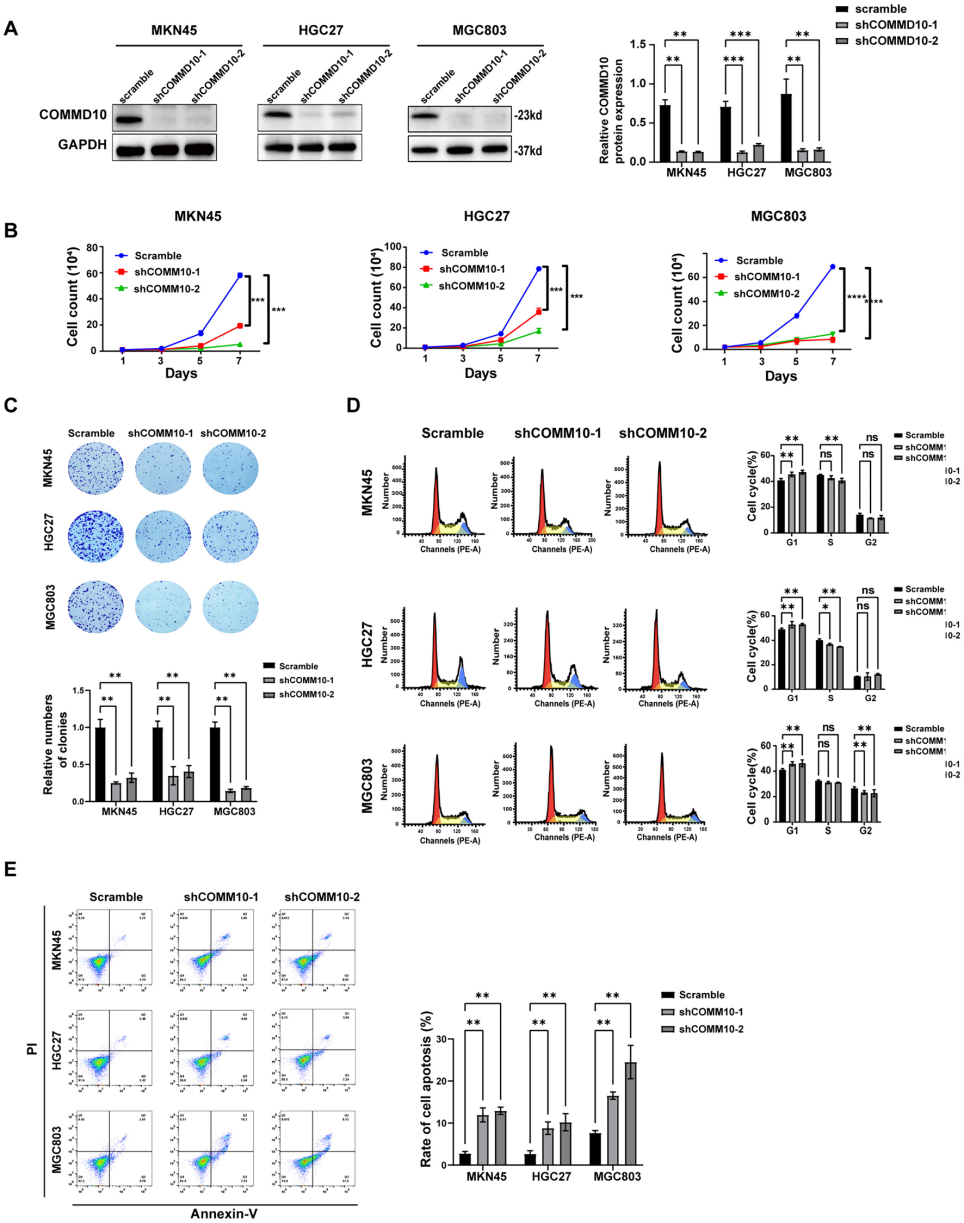
The Knockdown of COMMD10 reduced cell proliferation, promotes G1 phase arrest and increases apoptosis in GC. **A** Western blotting is used to verify the efficiency of COMMD10 knockdown. **B** Cell counting experiments and **C** colony formation experiments are conducted to show the effects of COMMD10 knockdown on the proliferation of GG cells MKN45, HGC27 and MGC803. **D** The cell cycle distributions and **E** apoptosis of COMMD10 knockdown in MKN45, HGC27 and MGC803 are analyzed by flow cytometry. *P < 0.05, **P < 0. 01, ***P < 0.001, ns indicates no statistical difference

To elucidate the function of COMMD10 on GC cells, we constructed two shRNA targeting COMMD10 and transfected them into the MKN45, HGC27, and MGC803 GC cell lines. The efficiency of COMMD10 knockdown was detected by qPCR (Fig. S2A) and western blotting (Fig. 2A). We found that COMDM10 knockdown markedly inhibited cell proliferation (Fig. 2B) and clone-forming ability (Fig. 2C) of the three GC cell lines. The EdU results also demonstrated that knockdown of COMMD10 suppressed the proliferation of GC cells (Fig. S2B). Besides, COMMD10 knockdown resulted in the inhibition of migration and invasion abilities in GC cells (Fig. S2C). Furthermore, flow cytometry showed that COMMD10 inhibition resulted in cell cycle arrest at the G1 phase (Fig. 2D) and significantly increased the apoptotic rate of GC cells (Fig. 2E). Therefore, these results confirmed that downregulation of COMMD10 inhibited proliferation, migration, and invasion, induced cell cycle arrest and increased the apoptosis rate in GC cells. In addition, the COMMD10 overexpression plasmids were transiently transfected into MKN45 and MGC803 cells (Fig. S3A). The results from cell proliferation assay (Fig. S3B) and clone formation assay (Fig. S3C) demonstrated that upregulation of COMMD10 significantly enhanced the capacities of proliferation and clone formation of gastric cancer cells. These results demonstrate that COMMD10 promotes the development of GC cells.

### The inhibition of COMMD10 causes the enhancement of DNA damage in GC cells

**Fig. 3:**
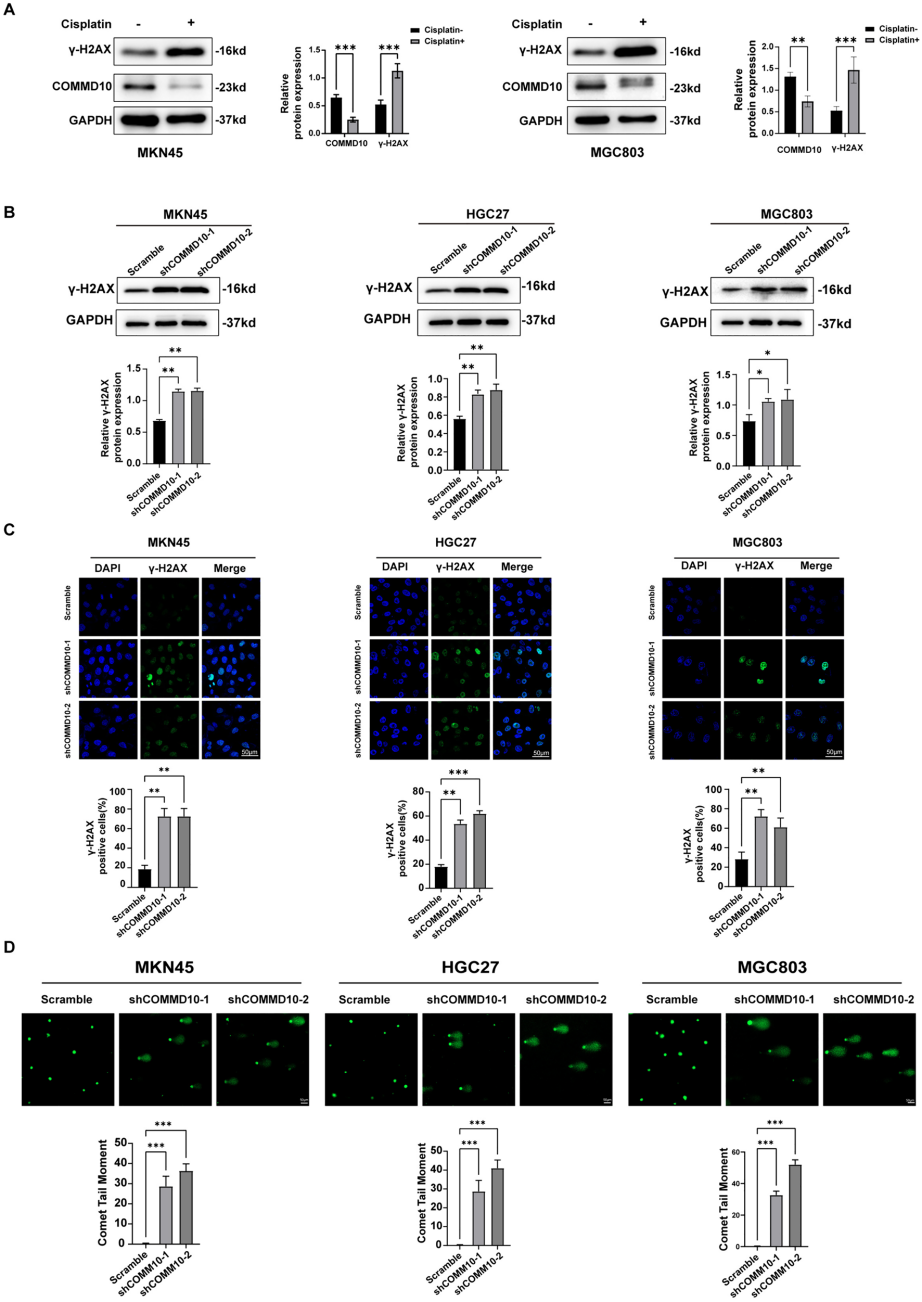
DNA damage is enhanced in COMMD10 knockdown GC cells. **A** The protein level of COMMD10 is reduced in cisplatin-treated MKN45 and MGC803 cells. **B** Western blotting and **C** immunofluorescence are performed to detect the expressions of DNA damage marker γH2AX in GC cells with COMMD10 knockdown. Scale bar: 50 μm. **D** The Comet assay is employed for the detection of DNA breaks. Scale bar: 50 μm. **P < 0. 01, ***P < 0.001

COMMD1 and COMMD4, members of the COMMD family, were involved in the process of DNA damage in cancer cells (Suraweera et al. 2021a, b). Combined with the finding that COMMD10 knockdown inhibited cell proliferation and led to cell cycle arrest in GC cells, we wondered whether COMMD10 also participates in the DNA damage process in GC. Cisplatin, a widely utilized chemotherapeutic agent for anti-tumor treatment, exerts its anti-tumor effect primarily by inducing cross-linking of DNA purine bases, thereby disrupting the DNA repair mechanism in cancer cells and promoting DNA damage and apoptosis (Romani 2022; Yimit et al. 2019). The western blotting results demonstrated that treatment of MKN45 and MGC803 cells with cisplatin for 24 h enhanced the expression of γ-H2AX levels, a DNA damage marker (Siddiqui et al. 2015), indicating the induction of DNA damage in gastric cancer cells by cisplatin. Interestingly, a reduction in the protein level of COMMD10 was also observed in DNA-damaged MKN45 and MGC803 cells (Fig. 3A), suggesting potential involvement of COMMD10 in the process of DNA damage in GC cells. To further elucidate the role of COMMD10 in DNA damage of GC. Western blotting (Fig. 3A) and immunofluorescence (Fig. 3B) experiments were performed and revealed a significant increase in the protein levels of γ-H2AX after COMMD10 knockdown. In addition, we conducted the comet assay, which is a single-cell gel electrophoresis experiment used to detect DNA strand breaks (Collins et al. 2023). The results showed that the comet tail substantially increased following COMMD10 knockdown (Fig. 3C), indicating the presence of DNA damage. These results suggest that the inhibition of COMMD10 causes DNA damage in GC cells.

### COMMD10 inhibits ATM-p53 signaling pathway and promotes DNA damage repair in GC

**Fig. 4:**
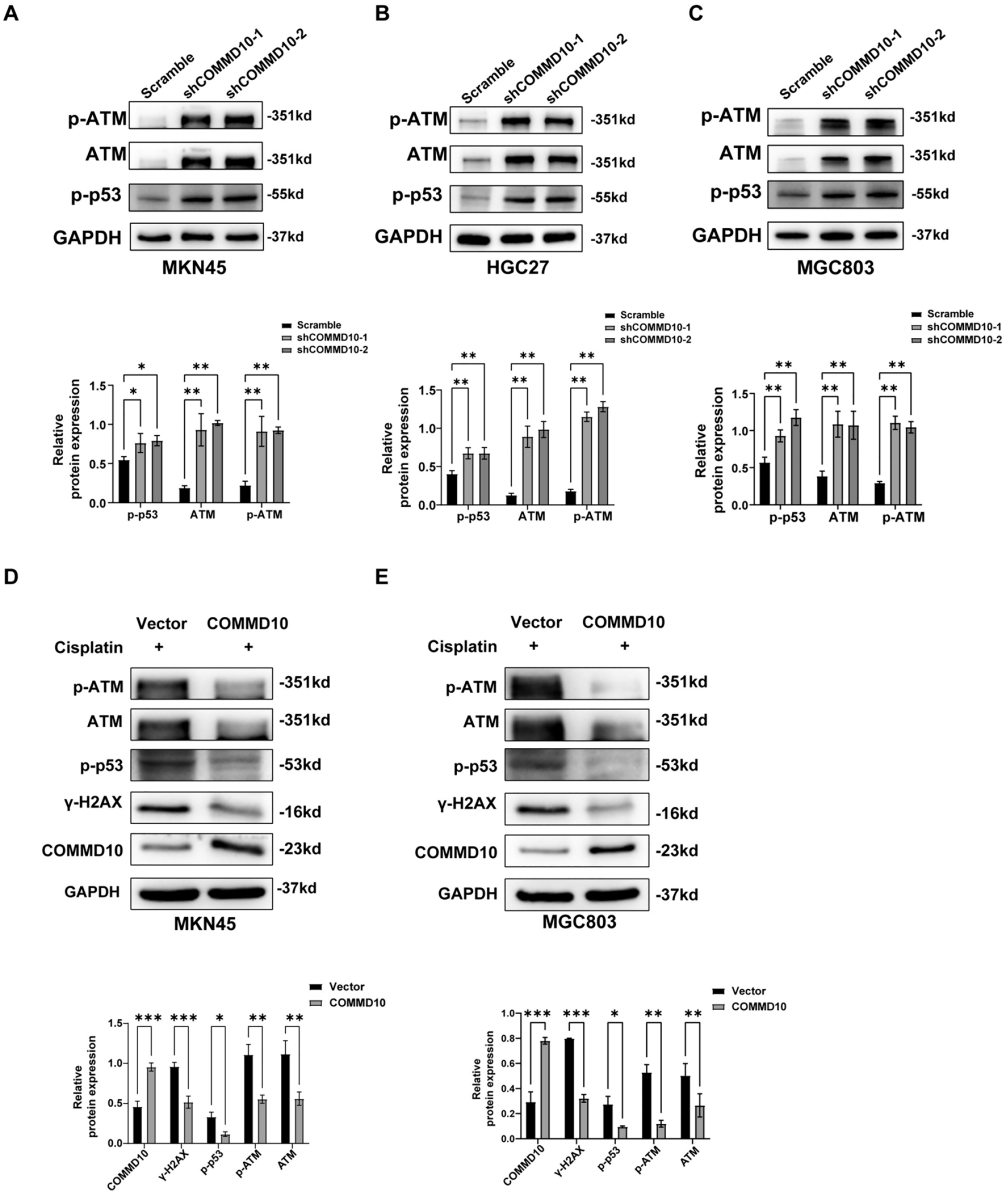
COMMD10 suppresses ATM-p53 pathway in GC cells. **A–C** Western blotting is conducted to detect the expression levels of DNA damage-related proteins including p-ATM (S1981), ATM, p-p53(ser15) in COMMD10 knockdown MKN45, HGC27, and MGC803 cells. **D, E** COMMD10 overexpression inhibits the expression levels of DNA damage-related proteins including γ-H2AX, p-ATM (S1981), ATM, and p-p53(ser15) in cisplatin-exposed MKN45 and MGC803 cells. *P < 0.05, **P < 0.01, ***P < 0.001

**Fig. 5:**
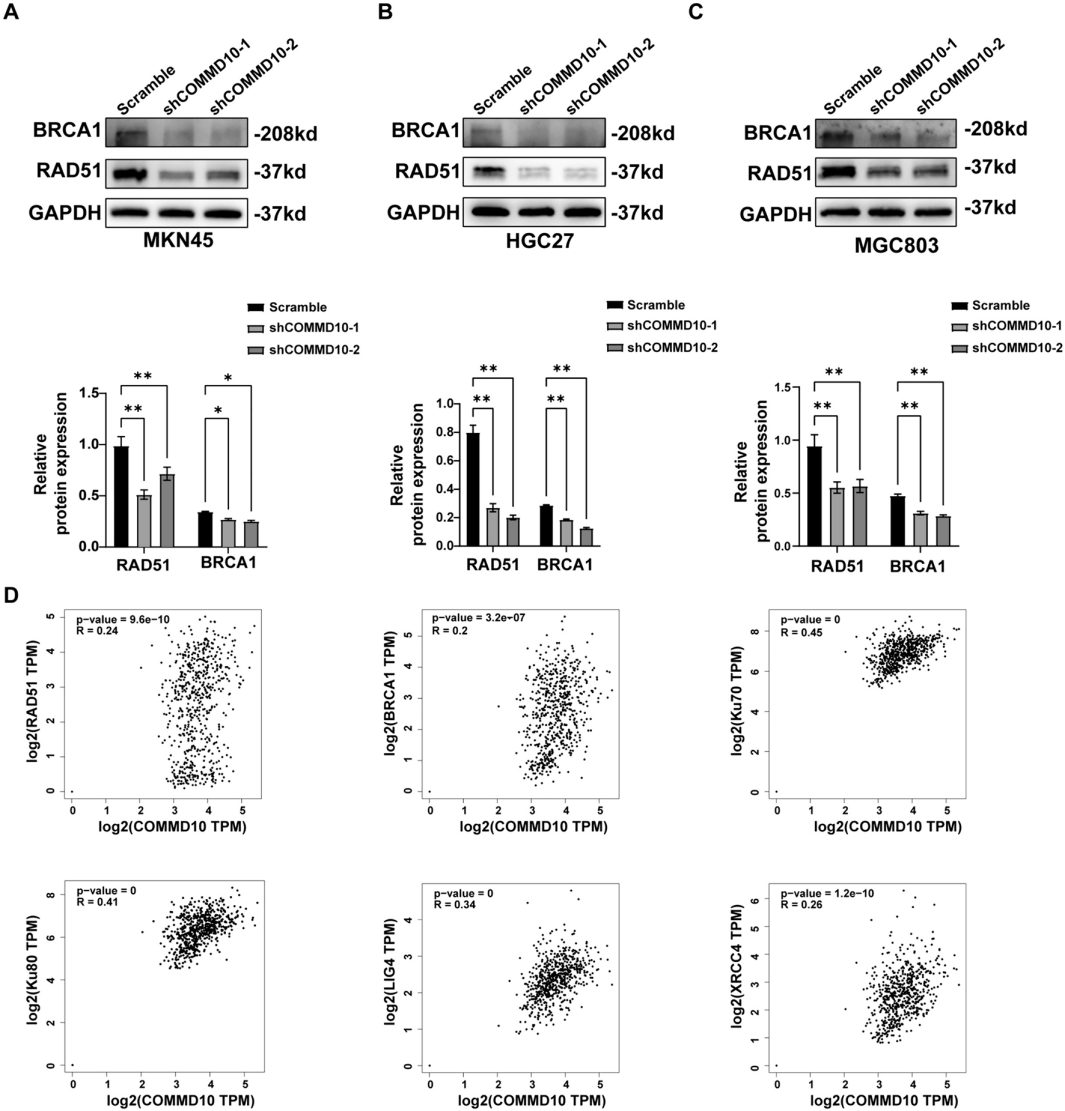
COMMD10 promotes DNA damage repair in GC cells. **A–C** The protein levels of DNA repair proteins RAD51 and BRCA1 are detected in COMMD10 knockdown MKN45, HGC27, and MGC803 cells. **D** The Expression correlations between COMMD10 and DNA repair proteins including RAD51, BRCA1, Ku70, Ku80, LIG4 and XRCC4 are investigated by GEPIA. R: Correlation coefficient*.* *P < 0.05, **P < 0.01. *P* ≤ 0.05 were regarded as statistically significant

In response to DNA damage signaling, the ATM protein is phosphorylated at Ser1981 and activates a series of downstream target proteins, among which p53 is an important protein that plays a crucial role in regulating the cell cycle, senescence, and apoptosis (Shiloh 2003; Lavin 2008; Bakkenist and Kastan 2003). Here, we detected the protein levels of ATM, p-ATM (Ser1981), and p-p53 (Ser15) after knockdown of COMMD10 and found that the levels of these proteins were significantly increased in the three COMMD10 knockdown GC cell lines. (Fig. 4A–C), While COMMD10 over-expression resulted in a decrease in the levels of γ-H2AX, ATM, p-ATM (Ser1981), and p-p53 (Ser15) that were enhanced by cisplatin-induced DNA damage in MKN45 and MGC803 cells (Fig. 4D, E). In addition, we restored the expression of COMMD10 in GC cells with COMMD10 knockdown and assessed DNA damage as well as protein expressions related to ATM-p53 signaling pathway. The results demonstrated that the restoration of COMMD10 reduced the expression levels of γ-H2AX, ATM, p-ATM (Ser1981), and p-p53 (Ser15) in MKN45 and MGC803 cells (Fig. S3D). These results demonstrated that supplementation of COMMD10 decreased the accumulation of DNA damage in GC cells, potentially attributed to the involvement of COMMD10 in DNA damaged repair. To investigate the role of COMMD10 in DNA damage repair, we assessed the expressions levels of critical DNA repair proteins BRCA1 and RAD51 in GC cells with COMMD10 knockdown. Western blotting analysis revealed a reduction in the expressions of BRCA1 and RAD51 following COMMD10 knockdown (Fig. 5A–C). To further elucidate the association between COMMD10 and DNA repair, GEPIA was employed to explore the expression correlations between COMMD10 and a panel of DNA repair proteins. The results demonstrated a positive correlation between the expression of COMMD10 and several key DNA repair proteins including RAD51, BRCA1, XRCC4, LIG4, Ku70, and Ku80 (Fig. 5D), which are involved in homologous recombination and non-homologous end joining(Khanna and Jackson 2001; Zhao et al. 2020). These findings indicates that COMMD10 plays a crucial role in DNA damage repair in GC cells, and its knockdown hinders this process, resulting in the accumulation of damaged DNA and activation of the ATM-p53 signaling pathway as a response to DNA damage.

### The knockdown of COMMD10 inhibits the tumor growth and exacerbates DNA damage in gastric xenograft

**Fig. 6:**
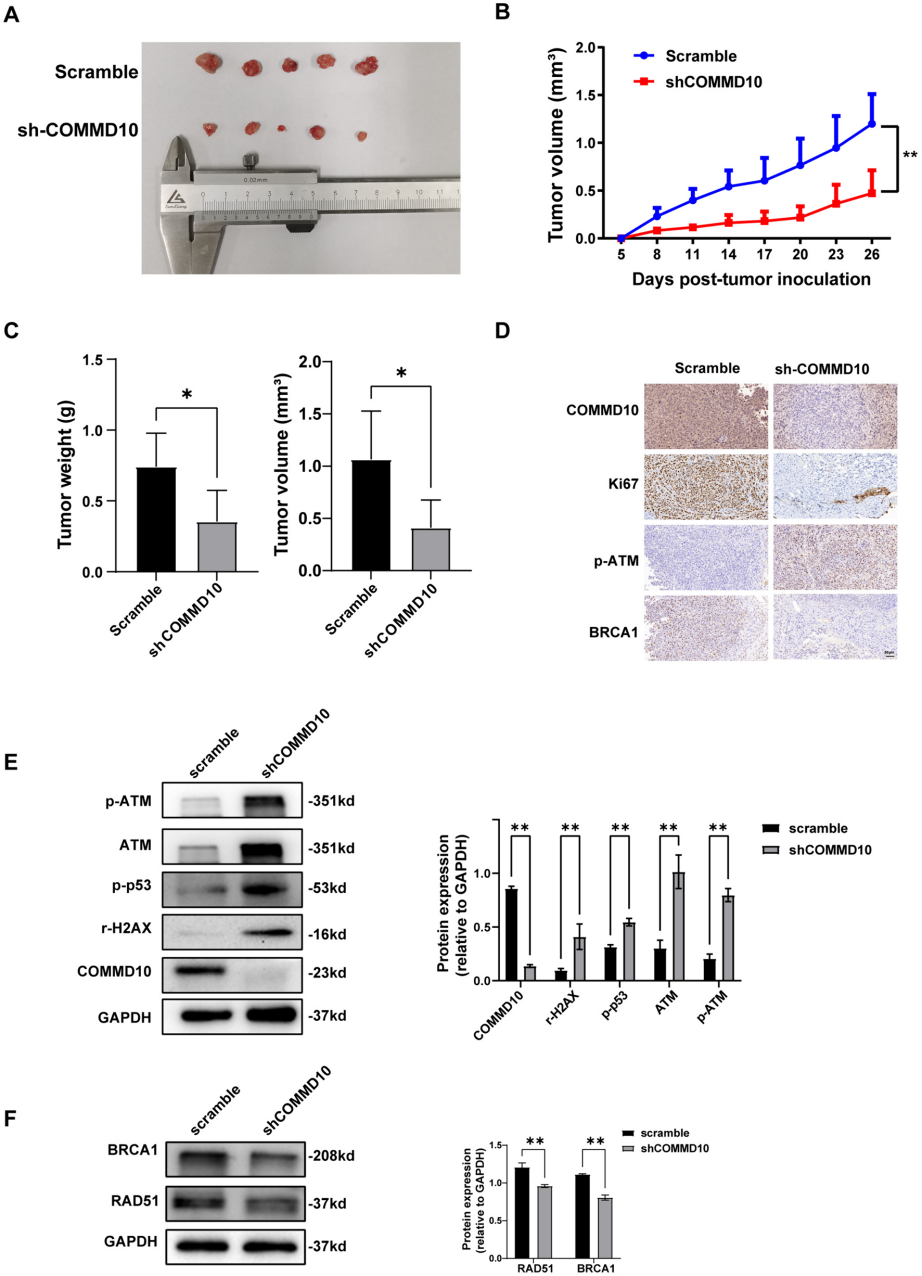
The Knockdown of COMMD10 suppresses tumor growth and exacerbates DNA damage in gastric xenograft. **A** Tumors are dissected and photographed after mice are sacrificed. **B** Tumor proliferation curve and **C** tumor volume as well as tumor weight in transplanted mouse model with COMMD10 knockdown are recorded and calculated. **D** IHC is performed to detect the expressions of COMMD10, Ki67, p-ATM and BRCA1 in tumors removed from mice. Scale bar, 50 μm. **E** Western blotting is used to detect the protein expressions of COMMD10 and DNA damage-related proteins p-ATM(S1981), ATM, p-p53(ser15) and γ-H2AX in xenograft tumor tissues with COMMD10 knockdown. **F** DNA repair proteins RAD51 and BRCA1 in xenograft tumor tissues with COMMD10 knockdown are detected by WB. *P < 0.05, **P < 0.01

A subcutaneous xenograft nude mouse model was established to verify the function of COMMD10 in GC cells. MKN45 cells transfected with scrambled shRNA or shCOMMD10 were injected subcutaneously into the axillae of nude mice. Xenograft tumor volume was monitored dynamically for 26 days. As shown in Fig. 6A, COMMD10 downregulation significantly suppressed the growth of xenograft tumor. Unsurprisingly, the tumor volume and weight were also reduced in the COMMD10 knockdown group (Fig. 6B, C). In addition, xenograft tumors were subjected to protein extraction and paraffin embedding for western blotting and IHC assays. As showed in Fig. 6D, IHC revealed higher expression of p-ATM (Ser1981) and lower expression of BRCA1 and ki67 in the COMMD10 knockdown group. Likewise, western blotting showed increased expression of p-ATM (Ser1981), ATM, p-p53 (Ser15) and γ-H2AX (Fig. 6E), whereas expression was decreased for the DNA damage repair proteins BRCA1 and RAD51 in the COMMD10 knockdown group (Fig. 6F). In conclusion, the down-regulation of COMMD10 results in the inhibition of GC xenograft growth, suppression of DNA damage repair, augmentation of DNA damage, and activation of the ATM-p53 signaling pathway in xenograft tumor tissues. These results are consistent with the outcomes observed in cellular experiments.

## Discussion

The COMMD family is involved in the development of various types of cancers, such as hepatocellular carcinoma (Zheng et al. 2019; Yang et al. 2021), non-small cell lung cancer (Suraweera et al. 2021a, 2020, 2021b; Zhan et al. 2017) and colorectal cancer (Yang et al. 2017). COMMD10 hinders the development of colorectal cancer (Yang et al. 2017) and hepatocellular carcinoma (Yang et al. 2021, 2022). However, whether the COMMD family plays a role in GC remains unclear.

In this study, we analyzed the expression and prognostic differences of the COMMD family in GC using the TCGA database and found that COMMD10 was highly expressed and associated with poor prognosis in GC. We further investigated the differential expression of COMMD10 in human GC tissues and obtained consistent results. We hypothesized that COMMD10 expression is involved in GC tumorigenesis and tested this hypothesis by knocking it down in three different GC cell lines. The functional experiments demonstrated that knockdown of COMMD10 suppressed the proliferation of GC cells, induced G1-phase cell cycle arrest, and facilitated apoptosis, while overexpression of COMMD10 promoted the proliferation in GC cells. Since the COMMD family members COMMD1 (Suraweera et al. 2021a) and COMMD4 (Suraweera et al. 2021b) had been reported to be involved in DNA damage repair to maintain the genome stability of the cancer cells, we wondered whether COMMD10 plays a role in DNA damage in GC cells. DNA damage can be induced by exogenous and endogenous factors such as ionizing radiation, genotoxic substances, reactive oxygen species, and replication fork stalling (Waterman et al. 2020). Cisplatin, a widely used chemotherapeutic agent, has proven to be effective in the treatment of various types of cancers. Its mechanism of action involves inducing DNA damage by forming cross-links with purine bases within the DNA molecule. This leads to the activation of the DNA damage response pathway, suppressing cell proliferation, ultimately exerting its anti-tumor effect in cancers (Romani 2022; Yimit et al. 2019). In our study, we observed an increase in the levels of the DNA damage marker γ-H2AX in MKN45 and MGC803 cells treated with cisplatin, indicating the accumulation of DNA damage. Interestingly, during the process of DNA damage in GC cells, there was a reduction in COMMD10 protein levels. Overexpression of COMMD10 mitigated cisplatin-induced DNA damage in GC cells. Furthermore, knockdown of COMMD10 resulted in the accumulation of DNA damage, which could be alleviated by restoration of COMMD10. These results suggest that COMMD10 is involved in the regulation of DNA damage in GC cells.

After DNA damage, DDR is activated, which causes cell cycle arrest and DNA repair. The most upstream DDR kinase is ATM, which recognizes DNA damage and is phosphorylated at Ser1981 to transmit a signal downstream (Shiloh 2003; Lavin 2008) One of the important downstream proteins is p53 that is phosphorylated at Ser15 in response to the DNA damage signal, leading to the inhibition of the cell cycle in the G1 phase and the initiation of apoptosis if DNA repair fails (Shiloh 2003; Bakkenist and Kastan 2003). To elucidate the regulatory mechanism of COMMD10 in DNA damage, we further investigated its impact on the ATM-p53 signaling pathway and observed an obvious increase in the protein levels of ATM and p-ATM (Ser1981) in COMMD10-knockdown GC cells. Moreover, the protein level of phosphorylated p53 (Ser15) was also increased in COMMD10-knockdown GC cells, which could explain G1 phase cell cycle arrest and increased apoptosis. Conversely, the overexpression of COMMD10 was found to suppress the expression levels of ATM, p-ATM (Ser1981), and p-p53 (Ser15) induced by cisplatin treatment, indicating that COMMD10 possessed inhibitory effects on DNA damage response in GC cells triggered by cisplatin. The restoration of COMMD10 expression in GC cells with COMMD10 knockdown decreased the protein levels of ATM-p53 signaling pathway caused by COMMD10 knockdown. Consequently, we hypothesized that COMMD10 may be implicated in DNA damage repair in GC cells. Subsequently, we detected the protein levels of RAD51 and BRCA1-two critical proteins involved in homologous recombination of DNA repair (Khanna and Jackson 2001) and found their reduced expression after the knockdown of COMMD10 in GC cells, indicating impaired homologous recombination repair for DNA damage in GC cells following COMMD10 knockdown. Homologous recombination and non-homologous end joining are the two main modes of repairing DNA DSBs, with non-homologous end joining being a major mechanism for DNA DSBs repair in higher eukaryotes. During the repair process of non-homologous end joining, the Ku70-Ku80 heterodimer first binds to the DSB and recruits other non-homologous end joining proteins. Direct ligation of DNA ends is accomplished by the LIG4 complex (Zhao et al. 2020). To further explore the relationship between COMMD10 and DNA repair, GEPIA was used to investigate the expression correlations between COMMD10 and RAD51, BRCA1, XRCC4, LIG4, Ku70, and Ku80, which are all DNA repair proteins involved in the process of homologous recombination and non-homologous end joining repair. The results showed positive expression correlations between COMMD10 and these DNA repair proteins, suggesting that COMMD10 was involved in these DNA repair processes, thereby promoting the repair of DNA damage in GC. The knockdown of COMMD10 impeded its reparative effects on DNA damage, resulting the accumulation of damaged DNA. Consequently, this triggered the activation of the ATM-p53 signaling pathway to regulate cellular fate. Moreover, the knockdown of COMMD10 suppressed the growth of GC xenograft tumors and hindered DNA repair while promoting DNA damage in transplanted tumor tissues. These findings aligned with the aforementioned cellular experiments.

COMMD1 has previously been shown to interact with BRCA1 and LIG4 (Woods et al. 2012), both of which are DNA repair proteins involved in non-homologous end joining and homologous recombination. Besides, COMMD4 maintains genomic stability by binding to histone H2B and protecting it from mono-ubiquitination by RNF20/RNF40 at DSB sites (Suraweera et al. 2021b). Combined with the results of bioinformatics analysis that COMMD10 was found to interact with COMMD4 and COMMD1 (Fan et al. 2020). We proposed that COMMD10 may have functions similar to those of COMMD1 and COMMD4 since they share a common COMMD domain, or work in a complex with COMMD1 or COMMD4 to regulate the DNA repair process in GC. Further studies are needed to confirm the detailed mechanism of COMMD10 in regulating DNA damage repair.

With the emergence of poly-ADP ribose polymerase inhibitors(Li et al. 2020), Targeting DNA repair proteins have become a hot spot for cancer treatment. Understanding the roles and associations of these DNA repair genes and proteins in DDR will contribute further clinical studies to the development of novel therapeutic strategies for GC (Wang and Xie 2022). Our finding that COMMD10 is involved in DNA damage repair in GC and protects GC cells from DNA damage implies that the inhibition of COMMD10 may be a new direction for GC therapy. However, the study has some limitations. The detailed mechanism by which COMMD10 regulates DNA damage repair in GC remains to be elucidated. In addition, the expression correlations between COMMD10 and DNA repair proteins were obtained from online databases, and more experimental data are needed for further validation.

In conclusion, we reported a novel role for COMMD10, which can promote DNA damage repair and maintain genome stability in GC. The knockdown of COMMD10 suppressed DNA repair, leading to increased DNA damage accumulation. Subsequently, the activated ATM-p53 signaling pathway induced cell apoptosis and inhibited cell proliferation, thereby impeding the development of GC. Targeting COMMD10 may offer a promising therapeutic approach for GC.

**Table 1:** Association of COMMD10 expression with clinical and pathological characteristics of GC patients

Features	N	COMMD10 expression		P value
		Low	High	
*All patients*	71	35	36	
*Age (years)*				0.2861
≥ 60	36	15	21	
< 60	35	20	15	
*Gender*				0.3956
Female	22	13	9	
Male	49	22	27	
*Depth of invasion*				0.1087
T1–T2	23	15	8	
T3–T4	48	20	28	
*Lymphatic node metastasis*				0.2795
No	25	15	10	
Yes	46	20	26	
*Distant metastasis*				0.6271
M0	67	34	33	
M1	4	1	3	
*TNM stage*				**0.0440**
I–II	33	21	12	
III–IV	38	14	24	
*Tumor size (cm)*				**0.0366**
≥ 5.0	28	9	19	
< 5.0	43	26	17	

*P*-values determined using chi-square test

Bold: *P* ≤ 0.05 were regarded as statistically significant

### Supplementary Information

Below is the link to the electronic supplementary material.

Fig S1. COMMD10 is correlated with poor prognosis in GC patients. **(A)** Disease-free survival, **(B)** Disease specific survival and **(C)** Progression free survival forest plot of COMMD family in GC patients (N = 375). **(D)** Disease-free survival, (E) Disease specific survival and **(F)** Progression free survival between COMMD10 high and low expression in GC through TCGA database. HR: hazard ratio, CI: Confidence Interval. P ≤ 0.05 is regarded as statistically significant (TIF 26935 KB)

Fig S2. COMMD10 knockdown inhibits proliferation, migration and invasion of GC cells. **(A)** qPCR is performed to validate the efficiency of COMMD10 knockdown in MKN45, HGC27 and MGC803 cells. **(B)** EdU assays are employed to explore the proliferation of COMMD10 knockdown MKN45, HGC27 and MGC803 cells. Scale bar: 20 μm. **(C)** Transwell experiments are performed to explore the abilities of migration and invasion of MKN45, HGC27, and MGC803 with COMMD10 knockdown. Scale bar: 100 μm, **P < 0.01 (TIF 28547 KB)

Fig S3. COMMD10 overexpression promotes cell proliferation and inhibits DNA damage in GC. **(A)** The overexpression efficiency of COMMD10 in MKN45 and MGC803 is verified through western blotting analysis. **(B)** Cell proliferation counting experiments are performed to assess the effects of COMMD10 overexpression on the ability of proliferation in MKN45 and MGC803 cells. **(C)** Colony formation experiments are conducted to show effects of COMMD10 overexpression on the abilities of colony formation in MKN45 and MGC803 cells. **(D)**. The restoration of COMMD10 expression partially reduces the protein levels of γ-H2AX, p-ATM (S1981), ATM, and p-p53(ser15) in GC cells. *P < 0.05, **P < 0.01, ***P < 0.001(TIF 28197 KB)

## Data Availability

The datasets used in this study are available from the corresponding authors (Aiping Le and Piaoping Hu) upon reasonable request.
