# Supplementary figures and images for: COMMD10 inhibited DNA damage to promote the progression of gastric cancer
Source: J Cancer Res Clin Oncol. 2024 Jun 13;150(6):305. doi: 10.1007/s00432-024-05817-z (PMC11176250; doi:10.1007/s00432-024-05817-z)

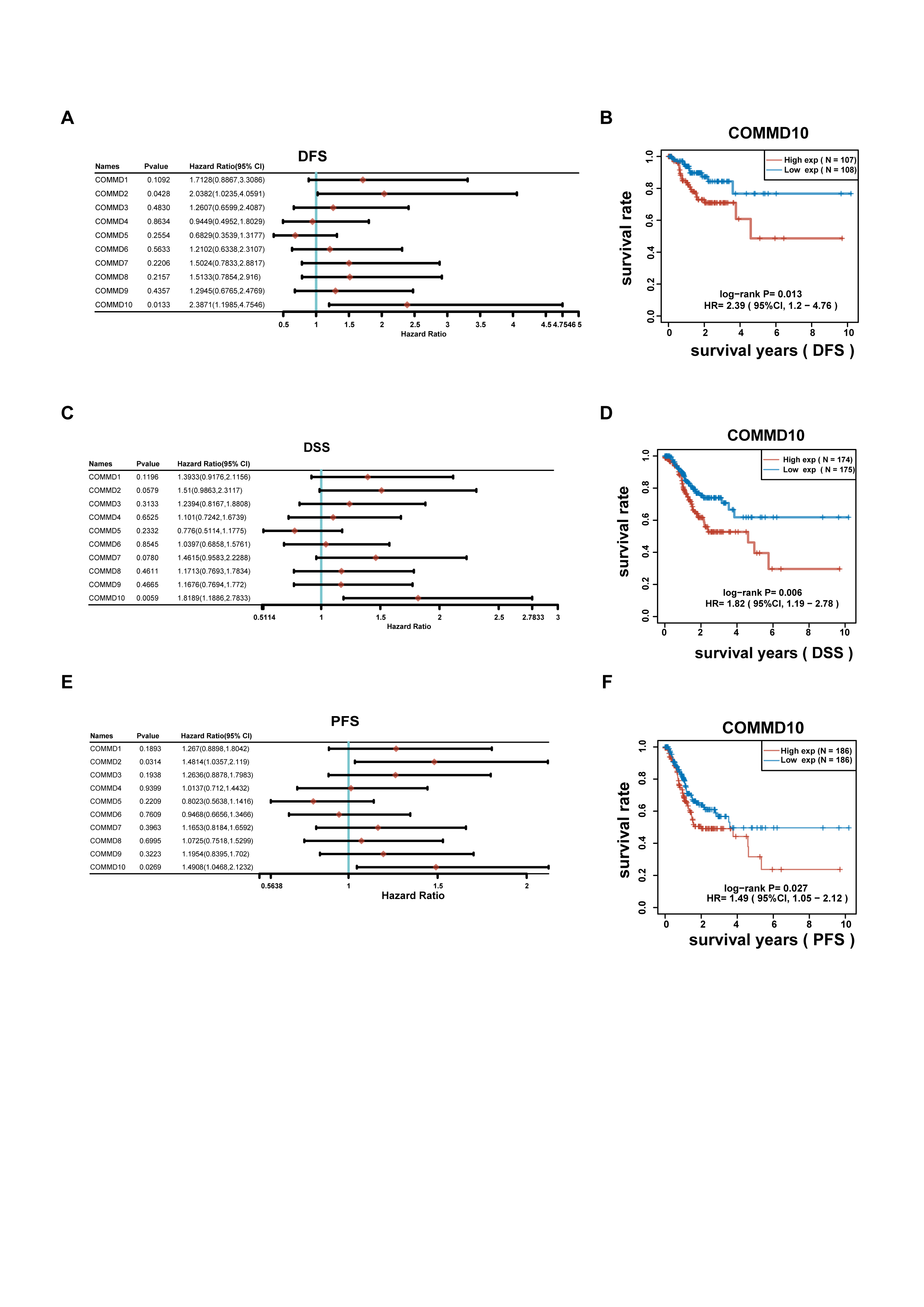

Supplement: Supplementary file 1 — Fig S1. COMMD10 is correlated with poor prognosis in GC patients. (A) Disease-free survival, (B) Disease specific survival and (C) Progression free survival forest plot of COMMD family in GC patients (N = 375). (D) Disease-free survival, (E) Disease specific survival and (F) Progression free survival between COMMD10 high and low expression in GC through TCGA database. HR: hazard ratio, CI: Confidence Interval. P ≤ 0.05 is regarded as statistically significant (TIF 26935 KB) [file 432_2024_5817_MOESM1_ESM.tif]

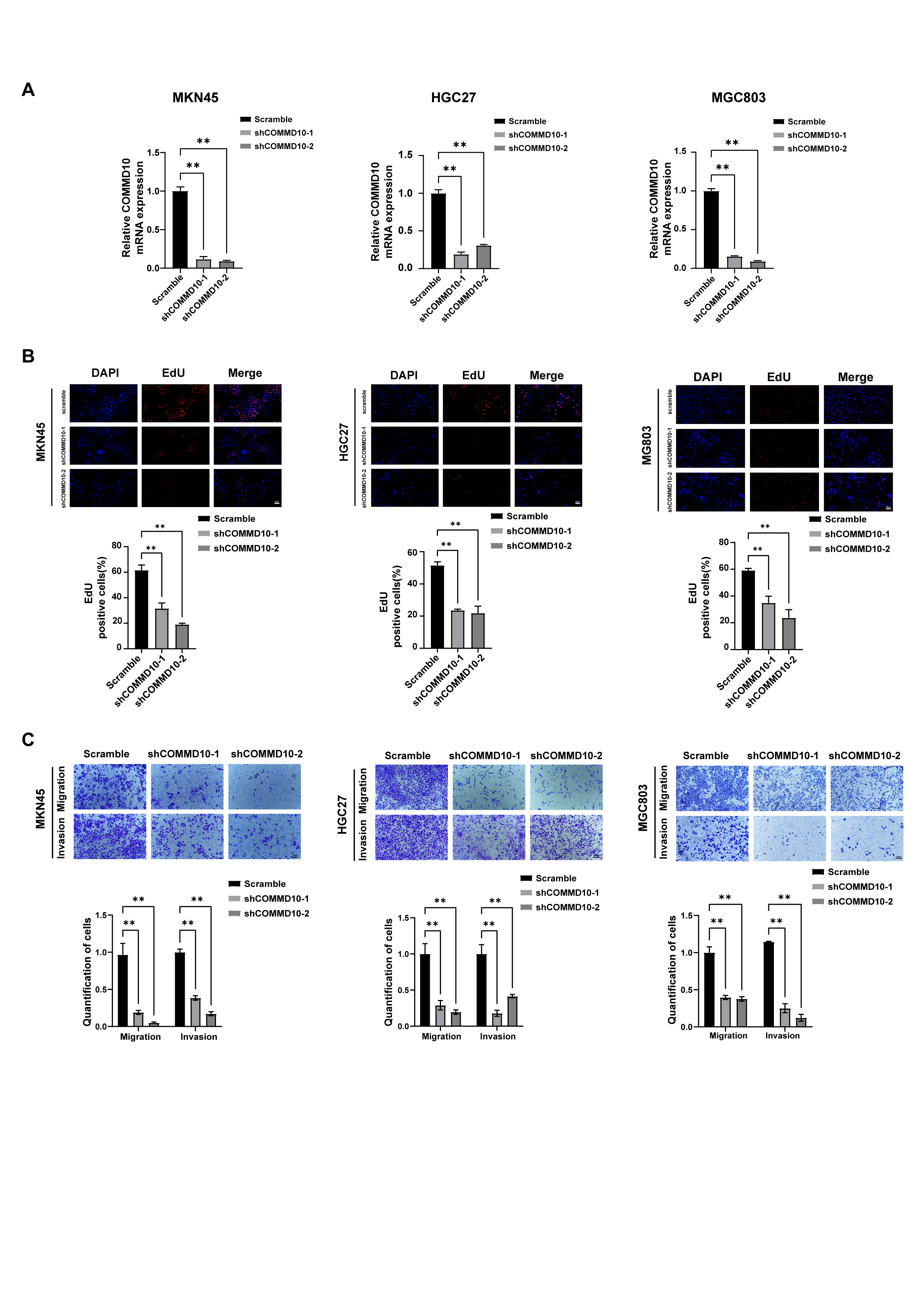

Supplement: Supplementary file 2 — Fig S2. COMMD10 knockdown inhibits proliferation, migration and invasion of GC cells. (A) qPCR is performed to validate the efficiency of COMMD10 knockdown in MKN45, HGC27 and MGC803 cells. (B) EdU assays are employed to explore the proliferation of COMMD10 knockdown MKN45, HGC27 and MGC803 cells. Scale bar: 20 μm. (C) Transwell experiments are performed to explore the abilities of migration and invasion of MKN45, HGC27, and MGC803 with COMMD10 knockdown. Scale bar: 100 μm, **P < 0.01 (TIF 28547 KB) [file 432_2024_5817_MOESM2_ESM.tif]

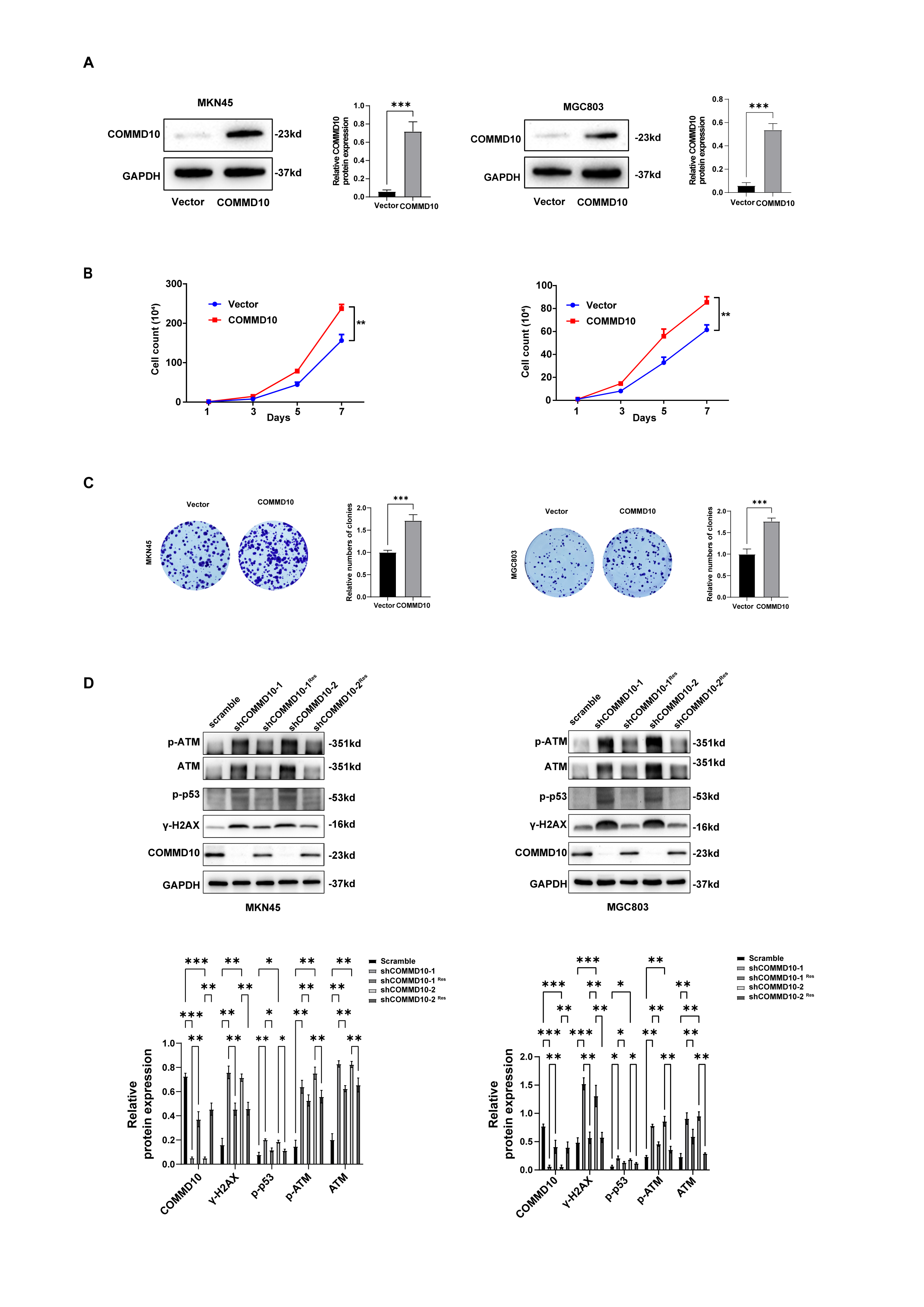

Supplement: Supplementary file 3 — Fig S3. COMMD10 overexpression promotes cell proliferation and inhibits DNA damage in GC. (A) The overexpression efficiency of COMMD10 in MKN45 and MGC803 is verified through western blotting analysis. (B) Cell proliferation counting experiments are performed to assess the effects of COMMD10 overexpression on the ability of proliferation in MKN45 and MGC803 cells. (C) Colony formation experiments are conducted to show effects of COMMD10 overexpression on the abilities of colony formation in MKN45 and MGC803 cells. (D). The restoration of COMMD10 expression partially reduces the protein levels of γ-H2AX, p-ATM (S1981), ATM, and p-p53(ser15) in GC cells. *P < 0.05, **P < 0.01, ***P < 0.001(TIF 28197 KB) [file 432_2024_5817_MOESM3_ESM.tif]
